# A Sisyphean task: experiences of perfectionism in patients with eating disorders

**DOI:** 10.1186/s40337-017-0136-4

**Published:** 2017-02-27

**Authors:** Suzanne Petersson, Per Johnsson, Kent-Inge Perseius

**Affiliations:** 1 Department of Psychology, Lund University, Lund, Sweden; 2 Department of Psychology, Lund University, Lund, Sweden; 3 The Swedish Red Cross University College, Stockholm, Sweden; 4 AnorexiBulimiCenter, Kalmar County Council, Kalmar, Sweden

**Keywords:** Eating disorders, Perfectionism, Qualitative research

## Abstract

**Background:**

Despite the theoretical links between eating disorders and perfectionism, the definition of perfectionism in practice is complicated. The present study explored descriptions and experiences of perfectionism described by a transdiagnostic sample of patients.

**Methods:**

In-depth, semi-structured interviews were carried out with 15 patients. The interviews were analyzed by Thematic Analysis. A comparison between the patients’ scorings on the Eating Disorder Inventory-Perfectionism scale was also performed.

**Results:**

Seven themes were found: The origins of perfectionism, Top performance, Order and self-control, A perfect body, Looking good in the eyes of others, A double-edged coping strategy, and A Sisyphean task. The women in this study did not emphasize weight and body as the main perfectionistic strivings. Core descriptions were instead order, self-control and top performances. All of the participants described the awareness of reaching perfectionism as impossible. Scorings of self-oriented perfectionism was significantly higher compared to socially prescribed perfectionism. No differences in the narratives related to perfectionism scores or eating disorder diagnoses were found.

**Conclusions:**

The results showed that psychometric measures do not always capture the patients’ definitions of perfectionism, but regarding that perfectionism serves as a means to regulate affects and may lead into an exacerbation of the eating disorder, and the development of obsessive-compulsive symptoms, it is important to investigate the personal definitions of perfectionism.

## Plain English summary

Perfectionism can lead to eating disorders, be a maintenance factor and interfere with treatment. In the present study we explored descriptions and experiences of perfectionism described by 15 patients, all women, treated for different eating disorder diagnoses. We interviewed the patients and compared their perfectionism scorings on an instrument constructed for assessing eating disorders symptoms. Seven themes were found; The origins of perfectionism, Top performance, Order and self-control, A perfect body, Looking good in the eyes of others, A double-edged coping strategy, and A Sisyphean task. The patients did not emphasize weight and body as the main perfectionistic strivings. Their core descriptions were order, self-control and top performances. Perfectionism was described to be a strategy to handle feelings of low self-esteem or anxiety. All patients considered perfection impossible and exhausting. Perfectionism was described to lead into a worsening of the eating disorder, and a development of obsessive-compulsive symptoms. We found no differences in the narratives related to perfectionism scores or ED diagnoses.

## Background

Perfectionism is one factor that, independently or in combinations with other risk factors, has shown to predispose and/or maintain ED symptoms in Anorexia Nervosa (AN), Bulimia Nervosa (BN) and Eating Disorder Not Otherwise Specified/Other Specified Feeding or Eating Disorder (EDNOS/OSFED) [1–4]. Perfectionism is a dichotomous concept, perfect or imperfect, which leads to inflexible standards. Negative events or actions get over-generalized and ruminated over, while positive events pass without much notice, or are rejected as too easy [5].

There are different conceptualizations and definitions of perfectionism [6]. In 1990 two research groups independently published measures of multidimensional aspects of perfectionism, sharing a common name, the Multidimensional Perfectionism scale, MPS [7–9]. The Frost MPS scale originally consisted of six dimensions of perfectionism and four of the subscales, Concern over mistakes, Doubt about actions, Parental Criticism, and Parental Expectations have been shown to be related to EDs [4]. The Hewitt & Flett MPS consists of three dimensions; Self-Oriented (SOP), Socially Prescribed (SPP) and Other Oriented (OOP) Perfectionism [8]. The combination of SPP and SOP, with differential roles, has been found to be associated with EDs [10, 11]. SOP has shown to be more strongly related to anorectic symptoms, while SPP has been found to correlate with negative affect, depression, and a wider range of EDs including BED [12–14]. In a study of patients with EDs, SPP was also found to be negatively correlated with Sense Of Coherence, especially with the meaningfulness and manageability components [15]. The Eating Disorder Inventory (EDI) contains a perfectionism subscale that has been shown to measure SOP and SPP aspects [11, 14].

Perfectionistic persons with low self-esteem base their self-worth on strivings towards perfect achievement and/or appearance [16]. Women with higher levels of perfectionism were shown to have the highest ED symptom levels [17]. Negative self-evaluation is common among those with an ED [18, 19]. Recent studies have shown that interactions between perfectionism and other risk factors rather than perfectionism alone have great impact on clinical impairment in persons with EDs. Self-critical perfectionism, combined with body dissatisfaction, has been shown to predict high levels of drive for thinness [20]. However, apparently impulsive behaviour, e.g. binge eating, has been shown to be related to self-critical perfectionism as well [21]. One explanation is that rigid control (dieting) might “spiral out”, and paradoxically lead to a loss of control, resulting in binge eating [22]. The combination of perfectionism and emotional dysregulation has been shown to predict ED related clinical impairment [23]. Self-criticism (Concern over mistakes), together with ineffectiveness, has shown to increase risk of ED development over time, while no support for a similar role for Personal standards perfectionism was shown [24].

Some aspects of the perfectionism concept have been described as normal/positive [25–27]. Two of the Frost MPS dimensions, Personal Standards and Organization, have been found to be positively correlated with positive affect and feelings of efficacy, and negatively correlated with frequency of procrastination, and have been suggested to measure a healthier aspect of perfectionism [7, 26]. Moreover, a study by Frost et al. [26] showed a positive relation between positive affect and SOP (measured with the Hewitt & Flett MPS), suggesting that SOP is a positive aspect of perfectionism. The positive and functional concept is considered to have little clinical relevance, and most research focuses on the dysfunctional aspects [28, 29]. Furthermore, SOP has been found to be related to eating disorder (ED) symptoms and can thus not be considered a positive aspect in that regard [4, 14].

There has been substantial research on perfectionism and EDs, mostly involving operationalized psychometric measures. When using such instruments in clinical assessment we feel they do not cover all facets of perfectionism as described by the patients, and furthermore, that there often appears to be a dissonance between the results of the assessments and the patients’ own descriptions. There is lack of systematized, detailed, and in-depth descriptions of perfectionism from a patient perspective. We believe that such descriptions, investigated by a qualitative approach, could help to refine future operationalization of the concept, as well as in the clinical situation, be helpful when addressing issues of perfectionism.

The aim of the present study was twofold: a) to illuminate experiences and descriptions of perfectionism in patients with EDs, and b) to investigate whether descriptions differed between participants with high or low scores, respectively, on a perfectionism measure.

## Method

### Study setting and clinical evaluation of perfectionism

The study was conducted at a public, youth/adult, integrated psychiatric outpatient clinic, the AnorexiBulimiCenter (ABC), specializing in the treatment of ED in southern Sweden. The clinic receives approximately 150 new patients yearly. About 2% of the patients are men. Diagnostic evaluation is performed at admission, by a staff experienced in the assessment and treatment of EDs, in a clinical interview, using the 36-item version of the Eating Disorder Examination Questionnaire, EDE-Q [30] and the Structured Eating Disorder Interview, SEDI [31]. The authors were not involved in the assessment or treatment of the participants.

The EDE-Q is a self-report measure focusing on behavioral features of EDs during the past 4 weeks. It contains four subscales (Restraint, Eating Concern, Shape Concern, and Weight Concern) as well as ratings of objective and subjective bingeing, purging, laxative/diuretic use and exercise [30]. The EDE-Q has shown good psychometric properties, including concurrent validity [32], internal consistency, temporal stability, and test–retest reliability [33].

The SEDI is a semi-structured interview based on DSM-IV and ICD-10 ED criteria and comprises 20–30 questions. Preliminary validation against the EDE interview has shown a concordance of 81% concerning specific ED diagnosis (including EDNOS and BED) and Kendall’ Tau-b of τ = .69 (*p* <. 0001) [31]. In this study the authors transferred the DSM-IV diagnoses to the DSM-5 criteria.

Upon admission patients filled in the Eating Disorder Inventory-3, EDI-3, although this was not part of the diagnostic battery, but used to guide treatment focus later on in the process. The EDI-3 is a self-report questionnaire designed to measure attitudes, personality features and ED symptoms. The instrument is internationally acknowledged, and consists of 12 primary scales, where one of the scales measures extent of perfectionism (Garner, [34]). The Perfectionism subscale has demonstrated high internal consistency as well as test retest reliability [34, 35]. The Perfectionism subscale can be divided into two separate subscales with three items each. One subscale is related to self-oriented perfectionism (EDI-SOP) according to the Hewitt & Flett MPS, while the other subscale is related to socially prescribed perfectionism (EDI-SPP) according to the same scale e.g. [14, 36].

### Participants

The sample was clinical and consisted of consecutive female patients who commenced treatment at the ABC and consented to participate in the study. At the time for inclusion no new male patients were registered at the clinic. Seventeen patients starting treatment during the second half of 2014 were asked to participate, two declined. The final sample consisted of 15 women ranging 18-44 years of age (Median = 24). The distribution of the DSM-5 ED diagnoses was: Anorexia Nervosa (AN: *n* = 4), Bulimia Nervosa (BN: *n* = 4) and Other Specified Feeding or Eating Disorder (OSFED: *n* = 7, including six participants diagnosed with atypical AN, and one with a low frequency BN). According to participants the age of onset for EDs ranged from 11 to 23 years (Median = 16 years). Seven participants had been previously treated for EDs. Five were being treated in a day care program at the unit at the time of the interview. Two were in treatment as inpatients at a psychiatric ward. The two youngest participants were in senior high school. All remaining 13 participants had completed senior high school, and three of them reported having a university degree, while another five were studying, or had studied for a short time at university. None of the participants were unemployed and four were on sick leave at the time of the interviews.

### Data collection

Qualitative data collection often takes the form of loosely or semi structured dialogue between a researcher and an informant in an interview with open-ended questions [37]. In the present study data was collected by individual semi-structured interviews. They were conducted according to an interview guide constructed by the authors. The foundation of the guide was based on methodlogical literature [37, 38] and on the authors’ experiences of performing qualitative research interviews [39, 40].

The interview guide used a funnel approach with open-ended questions, starting with a number of wider “trigger questions” for each area, such as “What role does perfectionism play in your life?”, or “What are the possible consequences of perfectionism?”, and “How do you think perfectionism develops”? Then the questions became more specific, such as “Would you call yourself perfectionistic?”, and “Are there any positive or negative effects of perfectionism?” The interview guide also included questions on definitions of perfectionism, variability versus stability, positive and/or negative aspects, and connections between perfectionism and health. During the process of developing the guide the questions were discussed with peer-clinicians and researchers. The guide was then tested in a series of three pilot interviews. Data from the pilot interviews was later included in the analysis, as the test resulted in only minor revisions of the original interview guide. The interviews were held at the treatment unit. They lasted between 45 and 70 min, and were audio recorded. The first author conducted all interviews.

### Data analysis

Data was analyzed by thematic analysis [41]. Thematic analysis is an experiential and theoretically flexible method compatible with different paradigms within psychology. “Thematic analysis can be a method that works both to reflect reality and to unpick or unravel the surface of ‘reality’” [41] p. 81. Themes can be larger or smaller regarding item content. However, the main issue is whether they capture something important in relation to the overall research question.

Data was transcribed verbatim by the first author and then analyzed as follows:

1. The transcripts were first divided into low and high perfectionism narratives (according to the informants’ scores on EDI-P), and were then further analyzed in these two datasets.
2. The first author read and reread the datasets.
3. The analysis began by open coding, where sentences and parts of sentences carrying meaning related to the aim of the study were recognized as initial codes.
4. The initial codes in the two datasets were then compared by the first (SP) and the last author (KIP), and as there were no apparent differences, the data was further analyzed as one dataset.
5. Codes from four of the interviews were then randomly drawn, and all three authors suggested, in an inductive process, preliminary themes for the initial codes.
6. Six themes were then agreed upon thus forming an initial theme system.
7. The codes from the following interviews were then either deductively fitted into the initial theme system or temporarily put aside as non-fitting codes.
8. The non-fitting codes were inductively used tracing new themes. Six additional preliminary themes were found.
9. All themes found were reviewed independently by the first and last authors in relation to codes and interview texts, and then discussed until consensus was achived. This process reduced the number of themes, and some themes were renamed. The theme system was then found consistent with the data and therefore regarded as final.

In order to enhance trustworthiness the first author kept a self-reflecting diary during the research process [38, 42]. The diary started with an analysis of pre understanding stemming from personal life experiences, work as a clinical psychologist, and from the research literature, and was then used for reflections over the entire research process; sampling, data collection, data analysis and interpretation of results. The diary was used in discussions between authors and in manuscript writing.

## Results

### The EDI-P scores

The results of the EDI-3 were checked during the data analyses to learn whether descriptions of perfectionism were correlated to the assessed degree of perfectionism. The EDI-3 cut off score, >7 points raw score according to Swedish norms [43] was used to determine whether a participant scored high or low on the EDI-P subscale. Five participants scored below (3–7 points), and ten scored above (8-24 points) the cut off score (Table 1).

**Table 1:** Eating Disorder Inventory-3 Perfectionism scale scores

Participants	EDI-P	EDI-SPP	EDI-SOP
1	5	1	4
2	24	12	12
3	13	3	10
4	3	0	3
5	8	4	4
6	6	2	4
7	19	11	8
8	11	2	9
9	14	4	10
10	15	5	10
11	19	7	12
12	22	10	12
13	8	4	4
14	7	0	7
15	4	1	3
Sum:	178	66	112
Mean (sd)	11.9 (6.8)	4.4 (3.9)	7.5 (3.5)
Range	3–24	0–12	3–12

EDI-P P scores (m = 11.9, sd. 6.8, range 3–24) were consistent with other Swedish samples of patients with EDs (m = 10.23, sd. 4.85) [35]. In the current study Cronbach’s α were: EDI-P: 0.87, EDI-SPP: 0.85, and EDI-SOP: 0.77. The Wilcoxon rank sum test was performed to compare the EDI-SPP and EDI-SOP scores. There was a significant difference (*p* < 0.008) between variables where the scores for SOP were higher. No differences in the narratives related to EDI-perfectionism scores or ED diagnoses were found.

### Themes

Seven themes were found in the data. Some themes were more categorical and others were more dimensional in their nature.

1. The origins of perfectionism
2. Top performance
3. Order and self-control
4. A perfect body
5. Looking good in the eyes of others
6. A double-edged coping strategy
7. A Sisyphean task

The authors’ clarifications in the quotations are written within brackets, while empty brackets stand for participant’s pause.

### The origins of perfectionism (*n* = 15)

Most participants described perfectionism as developing during childhood. The onset of perfectionism was most commonly regarded occurring at secondary school or 6th form. Some participants saw a connection between perfectionism and a growing bodily focus/awareness during secondary school (*n* = 2). Others underlined the pressures of academic achievement (i.e. grades) as causes for a perfectionistic development (*n* = 3). One participant considered perfectionism constitutional, while others described the development of perfectionism as a combination of constitution and environmental influences (*n* = 3).

*I think it’s sort of inborn, (…) it depends on how much you’re allowed to exercise it when growing up, whether those with whom you grow up show that you don’t have to be perfect, so you don’t have to follow those impulses, then you won’t become so perfectionistic when you grow up”* (Participant nr 2).

Parental influence was regarded as playing a considerable role according to participants, but theories of its mechanisms varied. Half the participants believed that demanding or high-achieving parents influenced the development of perfectionism, while others (*n* = 4) believed that careless or disorderly parents resulted in perfectionistic children.

*“You don’t become perfectionistic if you have perfectionistic parents”* (Participant nr 1).

Two participants grew up with seriously ill mothers with life threatening conditions. One of them theorized that this was related to her perfectionistic strivings; she felt she had to be an easy child so as not to trouble or burden her sick mother:

*“I think that it has always been like that because I didn’t want to be troublesome, and I didn’t want to be a burden to my mum,…while all the time striving to be the nice daughter as far as possible (…) I still think there’s a connection with wanting to please everybody, and make it easier for everyone, and not be in the way for anyone”. “I don’t think that you’re born with it but comes from something when you’re growing up”* (Participant nr 8).

Although perfectionism was, in one way or another, considered to originate from childhood and family, most participants that described themselves as perfectionists reported that their siblings were not perfectionists. Advertisements, media and social media were also indicated as mediators for development of perfectionism. A participant found hope in case there was a contextual learning development of perfectionism in a quote presented below:

*“In a way it would be better if it (*perfectionism*) was learned, then re-learning would be possible”* (Participant nr 9).

### Top performance (*n* = 15)

Top performance in different areas was one of the general definitions of perfectionism and reported by all participants. Top performance was experienced as demanded in school, at work, and in relationships. The latter was described in terms of the desire to be a perfect girlfriend, friend or daughter. Always striving for top performance was reported as tiresome. Some participants reported frustration for being close to perfection but felt they had “failed” even if they made but one mistake.

*“In my first year at 6th form I got top grades in all subjects except one, and…yes…I can still recall thinking” it would have been great to have the highest grade there too, and be able to say that I had top grades in all subjects since the first grade. I had only one mistake on all my vocabulary tests in all my school years, and since I only had one mistake, it’s pretty easy to remember”* (Participant nr 7).

Sometimes striving was planned to endure for just a short while, only for some goal that was considered reachable, and then rewarded with no further striving. Participants however, found it difficult to leave a perfectionistic strategy once initiated:

*“I thought that when leaving the 9th grade I would stop doing this and release control. And then I thought that it’d be nice to start in a new class (…) it started when people asked me* (about school stuff), *and thought that I was clever and asked me about this and that, returning again to my pursuit of perfection”* (Participant nr 14).

One participant used two strategies (competing above her level, or totally avoiding competition) in order to decrease pressure, as described below:

Regarding riding competitions: *“I compete somewhat over our capacity (…) all the time, then nobody has demands on us, then I make no demands on myself to win (but) just perform as well as I can. If I were to* (compete*) at my current level (…) then I’d rather skip the whole thing instead* (Participant nr 1).

### Order and self-control (*n* = 15)

Participants associated perfectionism with order and cleanliness. They talked of house cleaning, bed making, lawn cutting, and sorting of pens and books. They also spoke of cleaning up, not just their own homes, but their friends’ kitchens as well. According to one of the participants:

“*when I’m at someone’s home I can start cleaning up, and I don’t do that because I think that someone’s disgusting but I know that I do it fast (…) it’s like a skill I have (…) I can stare at the kitchen tile, it sort of distracts me, “good God how can I…can’t she go to the bathroom”* (so the participant can quickly clean the tile without being discovered) (Participant nr 11).

The function of order was described as a way of controlling oneself by controlling surroundings, as in the following description:

*“Inside my head I’m probably pretty untidy and then I compensate that by cleaning. That sounds really weird, I know”* (Participant nr 1).

### A perfect body (*n* = 8)

Some participants reported that their bodies were aims for their perfectionistic striving. The striving for better physical appearance as a part of perfectionism was pointed out in the latter parts of the interviews, and were considered to be the cause of ED development for these women. Participants described pressure from the social media to train and diet for fitness and achieve an ideal body. One participant had gained some weight and was influenced by others’ involvement in the pursuit of fitness as presented in the social media:

*“Everyone went to the gym and, well, lost a lot (*of weight*) and became gorgeous, and I went in the wrong direction. It was more than I could take”* (Participant nr 10).

A few participants considered Victoria’s Secret (VS) models, also called angels, as the ideal body image.

*“…you strive hard to look like a Victoria’s Secret model. They are super thin and fit”* (Participant nr 5).*“They (*the VS models*) radiate such self-confidence, and if I get as thin then I’ll get that self-confidence as well, and become as happy as they seem to be”* (Participant nr 7).

Physical training sometimes began with healthy intentions, and was considered a good hobby from the beginning according to participants. After a while the aim of the training changed. Some mentioned weight reduction as something initially positive, although they did not describe ideals or definitions of perfect bodies.

*“At first I thought it (*training*) was fun (…) but in the end it became mostly (…) looking like the ideal”* (Participant nr 8).

The down side of getting thinner and closer to the ideal body image was also discussed. The ideal body is considered to be both skinny and fit, which is contradictive. Exercising and increasing muscle mass on the one hand, and starving and having no body fat on the other was described as difficult.

*“It takes tremendous effort, particularly for girls, to get a six-pack. We’re not really made to have such a low lean-to-fat ratio (…). It looks great in a picture, but you have to put it in relation to what you’re willing to give”* (Participant nr 7).

While the participants quoted above tried to achieve an ideal body with diets and physical training, one participant dismissed this idea since she thought that a perfect appearance was:

*“to have natural features…to be naturally thin…*, *to not have to change much of yourself, to be natural. If you’re wearing makeup then you’re not perfect”* (Participant nr 4).

Although participants engaged in body shaping had a positive aim, one participant described how she experienced her skinny body as ugly during her worst anorectic period, and that she felt relieved when she had regained some weight and muscles.

*“…at the end (*of the ED*) I thought that I was actually sort of ugly, my ass hung and was all floppy. I really saw that at the end and I just felt shitty”* (Participant nr 6).

### Looking good in the eyes of others (*n* = 12)

Most participants reported the importance of gaining approval from others in relation to perfectionism. The need for approval was, however, described as troublesome. Participants were unsure about what and how much to do, and what was considered sufficient. They described that praise from others could initiate the desire for more praise, leading to a vicious circle of increased efforts toward enhanced and undefined goals.

*“I can get like that; now I’ve accomplished as much as possible, but what if people aren’t satisfied, or if it isn’t good enough? And then I do more” (*Participant nr 1).

A difficulty gaining positive attention for being oneself was described. A perfectionistic performance or appearance was suggested to be a way to project one’s personality into a more visible and concrete manifestation.

*“Maybe it’s hard to get validation eh…for yourself as a person but it’s easier to get* (validation) *for things that you have and things that are viewable rather than for who I am…inside”* and *“it’s not about myself, it’s about what you think others think about you in a way (Participant nr 15).*

Some participants had their own ideas of what people really thought about them, regardless of what those people said. They meant that there was no way to know the truth about others’ inner thoughts and opinions. Besides, praise from others was sometimes dismissed as something not to be trusted. They hypothesized that people may give praise just to make someone happy, no matter whether true or not, as suggested in a quotation below:

*“…they are parents, and you believe it’s their job to say such things so they aren’t necessarily…true”* (Participant nr 8).

Performing and behaving in a way that pleases others was considered important. Thus, this could turn up to be contradictory. One participant described how she stopped caring about significant others when it came to her anorectic behavior, which was a matter that really concerned and worried her closest friends and family.

*“…it’s really awful when I think about how I was this summer. I was really inside my own bubble, and I was just concerned with myself. I didn’t give a damn about others. Other people felt bad seeing me in that state, but I didn’t care and did just as I pleased*” (Participant nr 6).

Three participants emphasized that perfectionistic striving was self-imposed.

### A double-edged coping strategy (*n* = 15)

Perfectionism was on the one hand described in positive terms, and considered helpful in getting things done, getting organized, and gaining emotional control under trying circumstances. However, the dichotomous nature of perfectionism was also described as a harsh master when demands were not met. Furthermore, this coping strategy often fails in time. Distress becomes a problem for the individual.

“*I become depressed because I can’t keep things the way I want, and then I feel like a failure”* (Participant nr 9). *“I feel I’ve in some way failed, when things go wrong despite trying to get them right”* (Participant nr 8).

Comparisons with alcoholism (*n* = 3) were made on the basis of positive feelings when perfectionistic strivings succeeded. Participants described a connection between perfectionism and physical illness with bodies that hurt and stop functioning. They also reported connections between perfectionism and psychiatric disease, like EDs and Obsessive Compulsive Disorder, and associated their ED with control. The degree of perfectionism was considered to increase with the ED’s severity. Some of that control was reported related to treatment, with demands and rules for meals and eating but also directly to the illness as a reason for increased needs for control:

*“That’s why I became ill, I think, because then I could gain control over things”* (Participant nr 6). *“It’s much better when I’m not stuck in this eating disorder that I have. I wasn’t so perfectionistic when I felt good about things”* (Participant nr 3).*“Actually, it’s a high price to pay, being anorectic to avoid demands”* (Participant nr 12).

The risk of causing others to feel bad was also of concern. Maintaining strict order or high levels of success risked making friends feel bad.

Besides fearing the risk of hurting people close through perfection they were also afraid of arousing envy in others (*n* = 6).

### A Sisyphean task (*n* = 15)

All participants concluded that being perfect was unattainable, not just for themselves but for everyone. A few participants (*n* = 4) started the interviews by saying that they were not perfectionistic themselves, but after a short while all participants spoke from their own experiences and strivings. They described a problem in becoming perfect: is there no end?

*“The problem with perfectionism (*is that*) nothing’s perfect”* (Participant nr 2).

*“Sometimes I think I’m striving towards something I’m (…) not sure even exists”* (Participant nr 12).

Participants concluded that since the definitions of perfectionism differ from person to person, knowing when you have achieved perfection is impossible. They established that if a goal is reached, there is always another one beyond. Participants described how there were always limitations to their achievements, and that they, therefore, had to continue their efforts forever. In addition, others and life itself sometimes hampered perfectionistic intentions. Striving for perfectionism is a Sisyphean task. As two participants phrased it:

*“As soon as you reach one level it isn’t long before you strive to go farther”* (Participant nr 12).

*“It’s like running a race without ever reaching the finish line”* (Participant nr 9).

## Discussion

The present study explored experiences and descriptions of perfectionism from a patient perspective, in order to obtain detailed and in-depth information that could be used for future developments in operationalization of the concept, and to address perfectionism in clinical work with patients suffering from EDs. First of all, there were no differences in the narratives related to EDI-perfectionism scores or ED diagnoses, and the interviews were used as one dataset. The scores for SOP were significantly higher compared to SPP scores in the present study which corroborate earlier research (Petersson S, Clinton D, Brudin L, Perseius K-I: Perfectionism in eating disorders - are long-term outcomes influenced by changeability in initial perfectionism? Unpublished manuscript) [14, 35]. Higher levels of SOP have been shown in EDs compared to control or psychiatric groups, although various studies have shown different functions for SOP and SPP [4]. AM Lampard, SM Byrne, N McLean and A Fursland [14], for example, showed that SOP but not SPP was associated with dietary restraint, and weight and shape concern in EDs.

The origins of perfectionism have been shown to be multifactorial and transactional with interactions between psychological, environmental and biological vulnerability factors [44]. The participants’ descriptions of the development of EDs varied in similar ways as well. However, most participants felt that perfectionism developed during adolescence. The *Origins of perfectionism* theme was considered mainly related to familial factors, although interestingly, with conflicting hypotheses; either perfectionistic or “untidy” parents were considered the cause of perfectionism. This was in line with a study that found that both authoritarian (i.e. high on demandingness, low on responsiveness, controlling and evaluating), and indulgent (i.e. low on demandingness, high on responsiveness, and non-demanding) parenting styles seemed to foster perfectionism [45]. DR Hibbard and GE Walton [45] showed that combinations of parental styles and warmth in relation to their children either fostered or buffered maladaptive perfectionism, and either fostered or buffered adaptive perfectionism [45]. The participants in the current study, however, reported a difference between themselves and their siblings. They also reported that individual differences together with early messages from social media, coaches, peers, and the media, regarding achievement and success, may also influence the development of perfectionism. Social media is a relatively new phenomenon and studies have shown that idealized and hyperpersonal presentations are common, and when users of social media compare themselves with the idealized presentations this can lead to an increased dissatisfaction with their own lives and an increased pressure to live up to such hyperpersonal standards [46, 47].

Perfectionism was mainly associated with orderliness, cleanliness and top performance, described in *the Order and self-control and the Top performance* themes. These are concepts that relate to constructs such as “Conscientiousness”, “Organization” or “High standards” [6, 7, 48]. Researchers have not resolved if these concepts are to be considered parts of the perfectionism construct [6]. For example, RO Frost, P Marten, C Lahart and R Rosenblate [7] recommended that scores on the Organization subscales should *not* be included in the total score because of weak correlations with the other Frost MPS subscales. Adaptive perfectionism in some way reflects organization, conscientiousness, and an achievement–oriented style rather than perfectionism per se [48]. There could be another connection between conscientiousness and SOP. A longitudinal study on teenagers found that conscientiousness, as measured with the Neuroticism-Extraversion-Openness Inventory (NEO), predicted the development of SOP [49]. Notwithstanding the different theoretical conceptualizations of perfectionism, the participants emphasised the strong necessity of order, control and top-performance together with an experience that there was no end of struggle. Clinical perfectionism was suggested to be due to negative reinforcement (avoidance of negative affect/anxiety).

There are varying self-presentational styles. One style includes a need to appear perfect (to appear capable, competent and successful) in order to hide imperfections and perceived shortcomings to avoid criticism and maintain self-esteem [50]. This was illustrated by the quotation on competing above one’s level, thus decreasing possibilities to win, but providing, on the other hand, a possibility to explain less successful results. This might also be connected to the unwillingness of being associated with the term “perfectionism” mentioned in some of the interviews. To be perfectionistic might imply high demands, which should be avoided in order not to be exposed.

Appearance in the eyes of others (SPP) was held important by many of the participants, and was described in the *Looking good in the eyes of others* theme. However, EDI-scorings on the EDI-P showed a significantly higher extent of SOP than SPP among participants. The results of the EDI-P might depend on the items, since the EDI-SOP items relate to an all or nothing performance, being the best and setting high goals, while the EDI-SPP items relate to achievement directed toward parents/family and teachers. The women in this study did not express that their performance was particularly directed towards family or teachers. On the contrary, they expressed dubious thoughts about parental trustworthiness. Participants’ appearance concern was rather directed towards partners, peers, or workplaces. Hence, cultural and temporal differences might affect EDI-SPP ratings.

Human history is associated with periods of famine, but from the mid 20th century and onwards there has been better and more reliable access to food in western societies. When food intake is limited only by the individual’s ability to discipline, an equality of a thin-ideal and control has become possible [51]. Although research has shown that patients with an ED direct their perfectionistic striving to physical appearance [52], the aim of obtaining a perfect body was not described as a primary perfectionism target. Only half of the participants discussed perfectionism in relation to physical appearance. This suggests that the motive behind controlling the body is more often related to other functions, e.g. affect regulation, as suggested by F Skårderud and P Fonagy [53].

Perfectionism can be experienced as necessary when psychological well-being is affected, which was described in the *Order and self-control* and a *Double-edged coping strategy* themes. The downside of control is that harshness towards oneself and obsessive compulsive traits are enhanced at the cost of spontaneity. Some participants noted the risk of developing an obsessive compulsive disorder (OCD). Similarly, they associated their EDs with control. Emotional regulation deficits are common among persons with EDs [54]. Both EDs and perfectionism can provide a sense of order and control. Participants experienced that perfectionism increased along with the EDs. Perfectionism was considered secondary or contemporaneous, rather than predisposing, to the ED, which was contrary to their description of perfectionism in relation to OCD development, although in line with earlier findings on development of perfectionism in EDs [55].

### Methodological issues

The distinctive features and strengths of qualitative research is that it explores phenomena, reflects inter-subjective experiences, and can discover patterns from which hypotheses or operationalizations of concepts can emerge. A vital component for ensuring trustworthiness in qualitative research is the critical use of detailed descriptions of the phenomenon and the researchers familiarity with the phenomenon under investigation aas well as its culture, which we consider was met in the present study [38].

Investigation of results of the EDI-3 after the interviews, was performed with the intention of minimizing the influence of the self-report results on both the interviewer and participants. In order to enhance representation the interviewer made an effort to be a “naïve inquirer” with deepening and clarifying questions [42]. The authors were aware of, and considered the risk for, confusion between research and therapy when carrying out qualitative interviews [56].

The sample was clinical, and the informants were a representative group in this setting. Both high and low scorings of perfectionism (as measured by EDI-P), and various ED diagnoses were included in the sample, thus obtaining both a consensus *and* deviations from the material, making it more transferrable [57]. The participants were, however, relatively highly educated, and all were employed, which may have made the sample slightly high-achieving. The data collection was conducted in one single care setting, which might have biased the results and affected the transferability of the results negatively. Further the interviews were held on one single occasion. A series of interviews might have provided more in-depth material. Checking preliminary results with the respondents could have strengthened the dependability of the results (Patton, [38]). However, this was not chosen due to practical reasons.

## Conclusions

Although research has shown that patients with an ED direct their perfectionistic striving to physical appearance [52], the aim of obtaining a perfect body was not described as a primary perfectionism target in the present study. Some participants told of how the body project arose as an effect that started with healthy intentions. All participants described order and top performance as core descriptions of perfectionism, although this has been a challenged definition in research on perfectionism. There was a unanimous description of perfectionism as a toilsome and impossible task. The difficulty seems to be to stop a process that seems positive and advantageous through anxiety relief, control, and positive attention from others before the downsides become too imminent.

### Clinical implications

The results showed that measures like the EDI-P do not always capture a patient’s conception of perfectionism. Regarding that perfectionism may serve as a means to regulate affects, and may lead to an exacerbation of the ED, and to the development of obsessive-compulsive symptoms (which is an aggravating factor in ED development), an early exploration of personal definitions of perfectionism is important.

## Abbreviations

ABC: AnorexiBulimiCenter
AN: Anorexia Nervosa
BED: Binge Eating Disorder
BN: Bulimia Nervosa
DSM-IV: Diagnostic and Statistical Manual of Mental Disorders, fourth edition
ED: Eating disorder
EDE-Q: The Eating Disorder Examination Questionnaire
EDI: The Eating Disorder Inventory
EDI-P: The Perfectionism scale in the EDI
EDNOS: Eating Disorder not Otherwise Specified
ICD-10: International Classifications of Diseases, tenth edition
MPS: The Multidimensional Perfectionism scale
OOP: Other Oriented Perfectionism
OSFED: Other Specified Feeding or Eating Disorder
SEDI: The Structured Eating Disorder Interview
SOC: Sense of Coherence
SOP: Self-Oriented Perfectionism
SPP: Socially Prescribed Perfectionism
